# Fabrication of Carbon-Like, π-Conjugated Organic Layer on a Nano-Porous Silica Surface

**DOI:** 10.3390/nano10091882

**Published:** 2020-09-20

**Authors:** Hiroki Noguchi, Marzia Sultana, Nanami Hano, Yutaka Kuwahara, Makoto Takafuji, Shoji Nagaoka, Hongdeng Qiu, Hirotaka Ihara

**Affiliations:** 1 Department of Applied Chemistry and Biochemistry, Kumamoto University, 2-39-1 Kurokami, Chuo-ku, Kumamoto 860-8555, Japan; Hiroki_Noguchi@kumadai.jp (H.N.); marzia.shila@yahoo.com (M.S.); Nanami_Hano@kumadai.jp (N.H.); kuwahara@kumamoto-u.ac.jp (Y.K.); takafuji@kumamoto-u.ac.jp (M.T.); nagaoka@kumamoto-iri.jp (S.N.); 2 Department of Chemical Engineering, Jashore University of Science and Technology, Bangladesh, Churamonkathi-Chowgacha Road, Jessore 7408, Bangladesh; 3 Lanzhou Institute of Chemical Physics, Chinese Academy of Science, 18, Tianshui Middle Road, Lanzhou 730000, China; hongdeng2005@163.com; 4 National Institute of Technology, Okinawa College, 905 Henoko, Nago, Okinawa 905-2192, Japan

**Keywords:** amorphous carbon, black materials, organic-inorganic hybrid materials, π–π interaction, adsorbent, geometrical selectivity

## Abstract

This paper presents a new type of black organic material-porous silica composite providing an extremely highly selective adsorption surface. This black composite was prepared by lamination on nano-sized pores with a carbon-like, π-extended structure, which can be converted via the on-site polymerization of 1,5-dihydroxynaphthalene with a triazinane derivative and a thermally induced condensation reaction with denitrification. This bottom-up fabrication method on porous materials had the great advantage of maintaining the pore characteristics of a raw porous material, but also the resultant black surface exhibited an extremely high molecular-shape selectivity; for example, that for *trans*- and *cis-*stilbenes reached 14.0 with the black layer-laminated porous silica, whereas it was below 1.2 with simple hydrophobized silica.

## 1. Introduction

For a sustainable environment, there has been a universal requirement for selectivity enhancement in adsorbents in a wide range of research and industrial fields, and therefore the surface modification of porous carrier materials has been widely investigated. The dense immobilization of functional groups such as ionic or polar groups containing heteroatoms in porous carrier materials is one of the simplest ways to achieve this purpose. Further selectivity enhancement can be successfully observed in macrocyclic compound-immobilized adsorbents [1,2,3,4,5,6,7,8,9,10], whose high selectivity is achieved via the cyclic ordering of heteroatoms as interaction sources with a molecular pocket. However, owing to its complex synthetic process and difficult purification process, this approach has limited applicability in molecular design and synthesis. Nevertheless, selectivity enhancement has been achieved by non-macrocyclic linear compounds when a weak interaction site is integrated with highly oriented structures. For instance, significant selectivity enhancement is observed for structural isomers of polycyclic aromatic hydrocarbons when heteroatom-based non-ionic polar groups such as carbonyl units are integrated in polymer side chains that are in highly ordered states. Typical examples have been reported in long chain alkylated polyacrylate [11,12,13], alternating copolymer systems with phthalimides [14], polypeptide-based [15,16,17,18], and lipidic-ordered [19,20,21] organic phases. These successful results are because of the promotion of multiple carbonyl–π interactions [22,23] with guest molecules.

Other useful integrations and orientations of interaction sites can be obtained by π-electron-rich π-conjugated polymer systems. A π–π interaction is weaker than a carbonyl–π interaction [19,20,21,22,23], but π-electrons can be highly dense in a vast plane. Typical examples are graphene and its related materials. Therefore, various π-electron-rich adsorbents—such as aromatic side chain-containing polymers [24,25,26,27]; polycyclic aromatics, such as coronene and fullerene [28,29,30]; aromatic foldamers [31]; carbon nanotubes [32]; graphene [33,34,35,36]; and graphitic carbon—have been developed [37,38,39].

**Figure 1:**
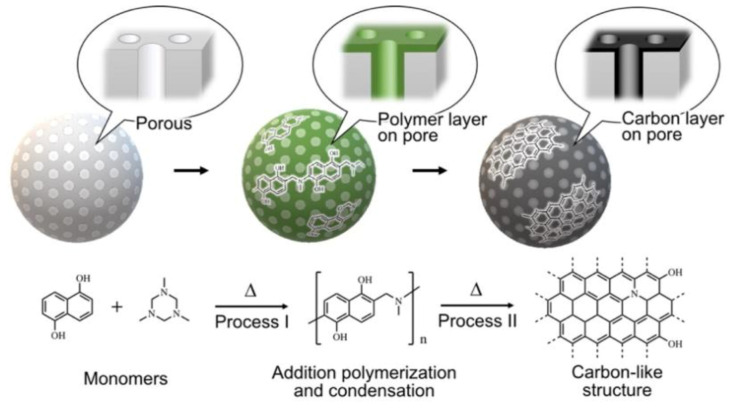
Schematic illustration of black layer-laminated porous silica through bottom-up carbon structure creation from 1,5-dihydroxynaphthalene (DHN) and 1,3,5-trimethyl-1,3,5-triazinane (TMTA), and its estimated reaction mechanism.

In this study, we present a facile and versatile fabrication method for the formation of π-electron-rich, carbon-like black organic phases on the surface of porous carrier particles. Our method can be emphasized by its distinct advantages compared with conventional carbon sources such as carbon black and graphite, which are the most readily available carbon sources, but their large size makes them unsuitable for the present purposes. Graphene is significantly small in thickness, and hence it can be used to wrap large surfaces. However, it cannot be used for coating at the nanosized inner interface of the pore [40]. Therefore, developing a bottom-up carbon structure creation method through which small molecules are adsorbed into the pores and that results in carbonization via post-treatment is necessary. Summarizing our method: (1) Long-ranged π-extended structures are created through the polymerization of small monomers and heat treatment, and then the resultant carbon-like layer can be fabricated on the surface of the nanosized pores in carrier particles (Figure 1). (2) The monomers used in this method are anionic and cationic. Therefore, the resultant carbon-like layer can be easily attached to various carrier surfaces via electrostatic interaction. (3) The degree of extension of the π-conjugated structure can be adjusted by the temperature and time of the heat treatment. Additionally, the adsorption ability can be tuned via this process. In this paper, we also demonstrate the strong absorptivity and high selectivity of the black layer-laminated porous silica as a new class of adsorbent.

## 2. Materials and Methods

### 2.1. Materials and Reagents

1,5-Dihydroxynaphthalene (DHN and 1,3,5-trimethyl-1,3,5-triazinane (TMTA) were purchased from Tokyo Chemical Industry Co., Ltd. (Tokyo, Japan). Ethanol, 2-propanol, and tetrahydrofuran (THF) solvents were purchased from Wako Pure Chemical Industry, Ltd. (Osaka, Japan). HPLC-grade acetonitrile, methanol, ethanol, and THF were purchased from Nacalai Tesque, Inc. (Kyoto, Japan). All the reagents were used without any purification. YMC SIL-120-S5 (YMC Co., Ltd., Kyoto, Japan, 5 μm diameter, 12 nm pore size, and specific surface area of 330 m^2^ g^−1^) were used as porous carrier materials. A commercial octadecylated silica (ODS) column (GL-science, Inertsil ODS-3, column size 150 × 4.6 mm i.d., with 5 μm particle size, 10 nm pore size, and 15% carbon loading) was used as the reference column.

### 2.2. Preparation of π-Conjugated Polymer-Silica Composites 

Process I with microwave heating: The procedure is as follows: 1 g of porous silica (YMC SIL-120-S5) and 8 mL of 2-propanol were mixed in the reaction vessel of the micro-wave synthesizer (Monowave 300, Anton Paar Gmbh, Graz, Austria) [41,42,43], and then the mixture was dispersed for 1 min using an ultrasonic bath (SONO CLEANER 100D, Kaijo Corporation, Osaka, Japan). Thereafter, 89.7 mg (0.56 mmol) of DHN and 72.4 mg (0.56 mmol) of TMTA were added to the dispersed mixture, and then the mixture underwent ultrasonication. The reaction vessel was placed in a micro-wave synthesizer and heated at 100 °C for 3 min while stirring. The obtained slurry was filtered, and the residue was thoroughly washed with 2-propanol and EtOH, and then dried in vacuo at room temperature. Process I without microwave heating: The procedure was as follows: 5 g of porous silica (YMC SIL-120-S5) was ultrasonically dispersed in 230 mL of EtOH in a 500 mL flask, and then 0.55 g (3.5 mmol) of DHN and 0.45 g (3.5 mmol) of TMTA were added to the dispersed silica. The mixture was gently stirred at room temperature for 1 min, heated to 75 °C, and then stored at 75 °C for 4 h. The obtained greenish product was filtered, and then the residue was thoroughly washed with EtOH and dried in vacuo at room temperature.

### 2.3. Blackening of Polymer-Silica Composites

The blackening of the polymer-silica composites, which corresponded to carbonization, was carried out in the presence and absence of a microwave synthesizer. Process II with microwave heating: a mixture of 2 g of the polymer-silica composites and 20 mL of ethylene glycol was redispersed in a reaction vessel of the microwave synthesizer, and then the mixture was heat-treated at 200 or 300 °C for 10 min in a microwave synthesizer. A dark-colored product was collected via filtration, and then it was thoroughly washed with EtOH and dried in vacuo at room temperature. Process II without microwave heating: 5 g of the polymer-silica composite was placed in a crucible (10 mL) and heat-treated in a nitrogen atmosphere at a predetermined temperature ranging from 200 to 900 °C for 2 h.

### 2.4. Instrumentations

The UV-visible and fluorescence spectra were measured using V560 (JASCO Co., Ltd., Tokyo, Japan) and FP-6500 (JASCO Co., Ltd., Tokyo, Japan), respectively. The reflective UV-vis spectroscopy was measured using USB4000 with a UV-vis-NIR light source DH-2000 and optical fiber (Ocean Optics, Largo, FL, USA). The obtained particles were observed via SEM Hitachi SU-8000 (Hitachi-Hightech Corp., Tokyo, Japan). Osmium tetroxide samples were treated using a Filgen osmium plasma coater OPC60A before observation. Thermogravimetric analysis (TGA) was conducted using the TG/DTA6200 (Seiko Instruments Inc., Chiba, Japan) in atmospheric air. The heating rate was set at 10 °C min^−1^. Elemental analyses were carried out on Yanaco CHN Corder MT-6 apparatus (YANACO Co., Ltd., Kyoto, Japan). Raman spectroscopy was measured using a laser Raman spectrophotometer NRS-5100 (JASCO Co., Ltd., Tokyo, Japan). The Raman spectra were recorded at a 532 nm laser excitation wavelength with a power of 5.5 mW. Each spectrum was recorded for 40 s with 10 accumulations and with a spectral resolution of 2 cm^–1^. The specific surface area and pore properties of the prepared particles were measured using NOVA2200 (Quantachrome, Boynton Beach, FL, USA) based on the Brunauer–Emmett–Teller (BET) and Barrett–Joyner–Halenda (BJH) methods. Liquid nitrogen was used as the coolant.

### 2.5. Evaluation of Absorptivity

The HPLC columns were obtained by packing the greenish and black-colored silicas in a stainless-steel column (150 × 4.6 mm i.d.). A 20 μL loop JASCO 980 pump with a Reodyne Model 7725 injector (JASCO Co., Ltd., Tokyo, Japan) and a JASCO MD-2010 plus UV–vis photodiode array detector (JASCO Co., Ltd., Tokyo, Japan) were used for the HPLC measurements. The column temperature was maintained using a column jacket with a heating and cooling system. A personal computer connected to the detector, and a pump with ChromNAV (Ver 1.17, JASCO Co., Ltd., Tokyo, Japan) software was used to control the system and analyze the data. Chromatography-grade solvents were used to prepare the mobile phase. The mobile phases used in HPLC were methanol–water mixtures, ethanol, and ethanol–THF mixtures. The separation factor (α) was obtained from the retention factor (*k*′) ratio. The retention time of water was used as the void volume (t_0_) marker (the absorption for methanol was measured at 220 nm). The water/1-octanol partition coefficient (log P) was determined using the retention factor with an ODS column: logP_o/w_ = 3.759 + 4.207log*k*′ (r^2^ = 0.99997) [44].

## 3. Results

### 3.1. Characterization of the Polymerization-Induced Assembly Method

**Figure 2:**
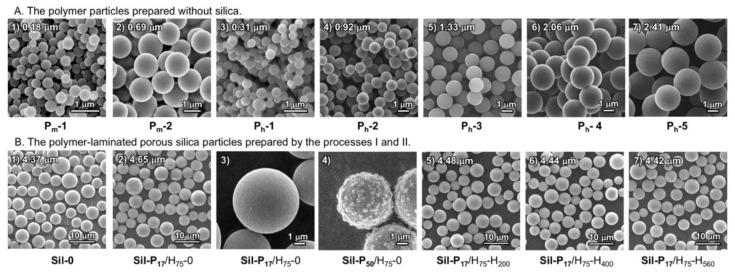
Scanning electron micrographs of the particles (**A**) without and (**B**) with silica, which were obtained by polymerization from DHN and TMTA. The size (μm) indicated in each photo corresponds to the average diameters of the particles. (**A**) The preparation condition is summarized in Table S1. The photos **A**-**1** and **A**-**2** show the particles prepared with microwave heating at 150 °C. The photos **A**-**3**, **A**-**4**, **A**-**5**, **A**-**6,** and **A**-**7** show the particles prepared without microwaves at 75 °C. (**B**) The preparation condition is summarized in Table 1. The photo B-1 is for the raw silica (**Sil-0**) before polymerization. The photos **B**-**2**, **B**-**3,** and **B**-**4** show the particles after polymerization with **Sil-0**. The photos **B**-**2** and **B**-**3** are for the comparison of the surface smoothness between **Sil-P_17_** and **Sil-P_50_**, respectively. The photos **B**-**5**, **B**-**6,** and **B**-**7** show the **Sil-P_17_**/H_75_-0 particles after heat treatment at 200 °C, 400 °C, and 560 °C, respectively.

To achieve a bottom-up carbon structure creation using small molecules in the inner pores, we applied the polymerization-induced self-assembling approach using hydroxyaromatic as a key monomer with a triazinane derivative as a cross-linker, which was developed to produce monodisperse nano-sized to micro-sized spherical particles [42,45]. Because of its adjustability on the assembling size and easy carbonization via simple heat treatment at a relatively low temperature, this approach is relevant to the present purpose. In this study, 1,5-dihydroxynaphthalene (DHN) was selected as a key monomer, and the polymerization was initiated by heating a DHN-containing solution in the presence of one equimolar of 1,3,5-trimethyl-1,3,5-triazinane (TMTA). As shown in the scanning electron microscope (SEM) of Figure 2A, the well-size-controlled spherical solid particles were produced spontaneously without any surfactant when a proper solvent such as ethanol and similar polar media (Table 1) were selected.

**Figure 3:**
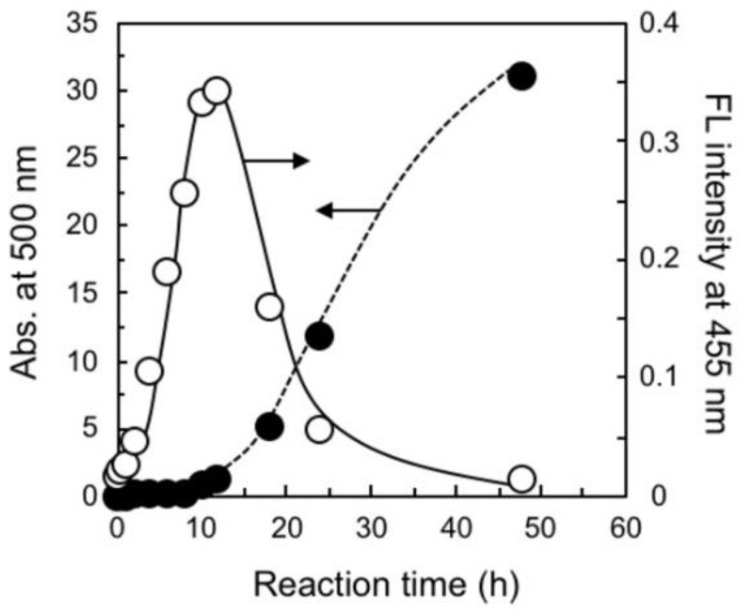
Time courses of the absorbance increase (solid circles) at 500 nm and the fluorescence change (open circles) at 455 nm upon excitation at 400 nm in the polymerization of DHN with TMTA. The polymerization condition is summarized in **P_h_-1** of Table 1.

**Figure 4:**
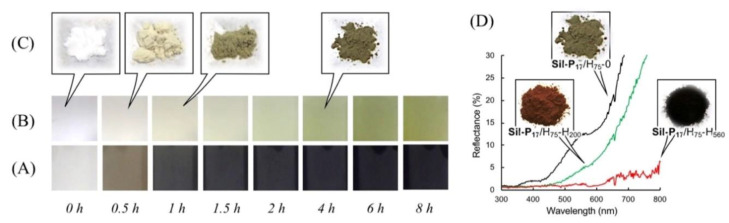
Monitoring of the color change during the polymer lamination process. The polymerization was carried out using 1 g of **Sil-0**, 120 mg of DHN, and 97 mg of TMTA in ethanol at 75 °C. (**A**) and (**B**) show the color difference of the sample suspensions without dilution and with dilution to 1/10, respectively. (**C**) shows the color difference of the corrected solid. (**D**) shows the reflection spectra of the corrected solid.

**Figure 5:**
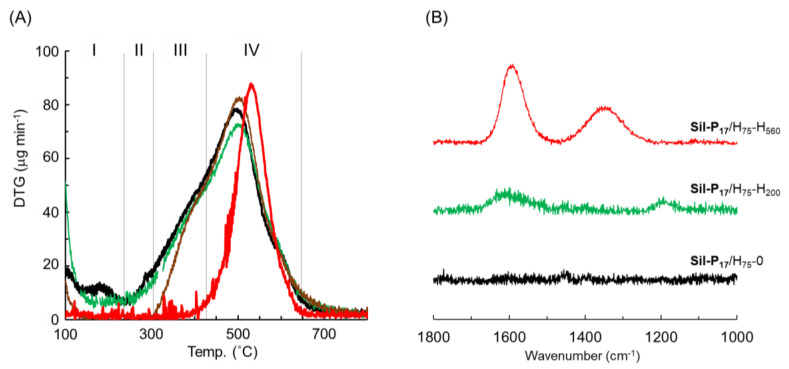
Characterization of the heat-treated **Sil-P_17_**. (**A**) Derivative thermogravimetric profiles of **Sil-P_17_**/H_75_-0 (**––**), **Sil-P_17_**/H_75_-H_200_ (**––**), **Sil-P_17_**/H_75_-H_400_ (**––**), and **Sil-P_17_**/H_75_-H_560_ (**––**) in air. (**B**) Raman spectra of **Sil-P_17_**/H_75_-0 (**––**), **Sil-P_17_**/H_75_-H_200_ (**––**), and **Sil-P_17_**/H_75_-H_560_ (**––**).

**Figure 6:**
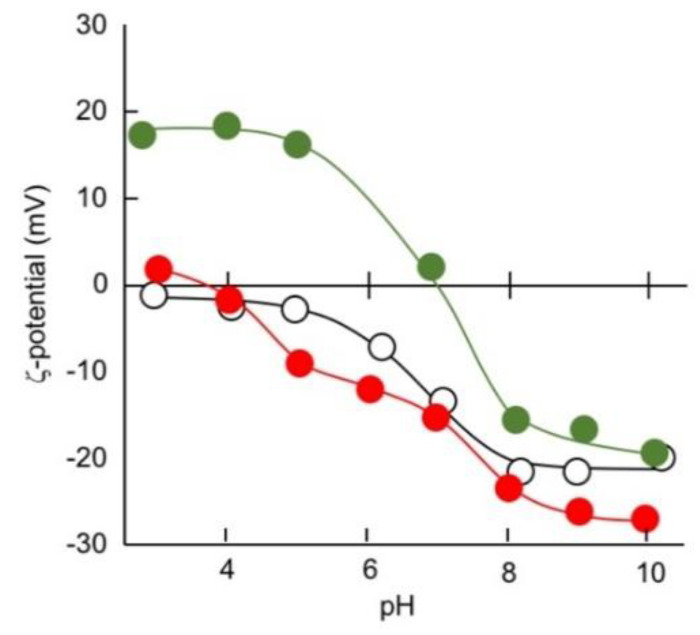
pH dependencies of the ζ-potential of **Sil-0** (**––**), **Sil-P_17_**/H_75_-0 (**––**), and **Sil-P_17_**/H_75_-H_560_ (**––**).

The polymerization conditions are summarized in Table 1. Figure 2A-1,2 show the SEM images from the particles prepared with microwave heating at 150 °C, and the SEM images in Figure 2A-3,4,5,6,7 were from the particles prepared without microwave heating at 75 °C. The polymerization rate was significantly accelerated by microwave heating. However, a conventional batch method without microwave heating was useful for large-scale production. The SEM images showed that the obtained solids exhibited a spherical shape with a good monodispersity regardless of the heating methods. In addition, all the particles were insoluble and less swelled in any solvent presumed to be a high-density crosslinked resin-type polymer. Therefore, it is estimated that the polymerization-induced aggregation is accompanied by a cross-linking reaction for solidification.

Through the dynamic light scattering (DLS) method, the polymerization progress was monitored by the particle diameter. Figure S1 shows the typical time courses of the increase in the particle diameter during polymerization. Considering this result and the fact that the particle diameter can be affected by the reaction conditions (Table 1 and Figure 2A), various factors can be expected to play a key role in the laminating process on a pore surface with a polymer layer.

To estimate the reaction mechanism of this polymerization, the time course of the carbon and nitrogen content ratio (CN ratio) in elemental analysis was examined without microwave heating. The CN ratio increased from 8.5 to 10.0 at a time course ranging between 6 and 8 h; the detailed data are given in Figure S2. These values are within 6.8 and 11.1, which were calculated as one-to-two and one-to-one addition products (whose structures are estimated in Figure S2), respectively. In contrast, we detected a significant increase in fluorescence at the initial stage. Figure 3 contains the time courses of the fluorescent intensity at the emission maximum at 455 nm upon excitation at 400 nm and the absorption increase at 500 nm when the reaction was monitored at a low concentration condition of 1/30 to reduce the reaction rate; the detailed spectra are given in Figure S3.

The **P_m_** and **P_h_** particles were prepared with and without microwave heating, respectively. The concentration indicates that of each monomer. The diameter and CV were determined by a SEM analysis of the corrected solids after the proper washing and drying processes.

No similar emission was observed in the raw material DHN. Finally, the reaction mixture changed to a non-fluorescent and dark-colored suspension as the reaction progressed. These results indicate that the fluorescent species are formed as an intermediate during polymerization, resulting in a π-extended structure. According to the literature [46], the cross-linking polymerization of DHN has been obtained by mixing ammonia with formaldehyde and not TMTA. Additionally, it explains that polymerization can be performed via the formation of the naphthoxide precursor (given in Figure S2), although it is not mentioned that fluorescent species are generated. Therefore, we estimate that our polymerization system includes a similar precursor formation with a fluorescent structure.

### 3.2. Formation of a Carbon-Like Black Layer on Porous Carrier

Based on the fundamental observations in the polymerization progress, we carried out this polymerization in the presence of porous silica (5 μm and 12 nm in average diameter and pore size; specific surface area of 330 m^2^ g^−1^ in the catalog specifications), which had been mostly used as carrier particles for high-performance liquid chromatography (HPLC). Table 2 summarizes the preparation conditions with the abbreviation of the products obtained.

The change in color during polymerization can be used to estimate the reaction progress. As shown in Figure 4, the white suspension owing to raw silica turned greenish and then deeply dark. Figure 2B-2,3 show SEM images of the colored silica (abbreviated as **Sil-P_17_**/H_75_-0), obtained using 20 wt % of DHN of silica. Compared to the raw silica (Figure 2B-1), the colored silica maintained a good sphericity, with a smooth surface after polymerization (Figure 2B-3). Additionally, the average diameter slightly increased by approximately 9%. In contrary, non-uniform surfaces (Figure 2B-4) were observed when approximately 50 wt % monomers were used in the polymerization process. This indicates that the excessive use of monomers promotes the undesirable self-growth of polymer particles on the silica surface.

In addition, owing to successive washing with various solvents such as ethanol, DMF, and chloroform, the colored silica had neither a significant color change nor reduction in the organic components. Therefore, it can be established that the polymer from DHN and TMTA was formed and tightly attached to the silica surface. This is probably due to the electrostatic interaction between the TMTA-derived imino groups of the polymer and the silanol groups of silica.

As shown in Figure S4, thermogravimetric analysis (TGA) of the colored silica (**Sil-P_17_**/H_75_-0) indicated that the amount of organic component attached on silica was ca. 15.2 wt %, considering the loss from silica because of the heat treatment. This value corresponds to 99% of the initially added DHN being attached as a polymer when it was assumed that the DHN was converted to a one-to-one addition polymer. The progress of such highly efficient polymerization also indicates that the cationic TMTA is effectively concentrated on the pore surface of the silica interface due to the electrostatic interaction. However, the slight elution of brown substances was observed by washing, while the polymer-coated silica did not change the color. This may be due to the by-production of a small polymer during the microwave heating, but this is not suitable for further application with HPLC because HPLC is very sensitive to such a slight elution. Therefore, we used only the polymer-coated silica obtained without microwaves for the HPLC study.

### 3.3. Blackening and Carbonization of the Polymer Component

The significant color changed from dark green to complete black when **Sil-P_17_**/H_75_-0 was incubated at higher temperatures, such as 200, 400, and 560 °C, under a nitrogen atmosphere. As shown in Figure 4d, **Sil-P_17_**/H_75_-H_560_ did not show any reflection in the visible light range. In addition, a further weight reduction was observed from 14.7 to 8.6 wt % (see Figure S4). Consistent with this result, Figure 2B indicates that the particle size gradually decreased with the increase in the incubation temperature.

The derivative thermogravimetric analyses (Figure 5a) of the TGA profiles provide important information on the weight loss leading to the blackening process. Figure 5a shows that the decomposition profiles of the **Sil-P_17_**/H_75_-H_200_ and **Sil-P_17_**/H_75_-H_400_ prepared via incubation at 200 °C and 400 °C were similar to that without additional heat treatment. The thermal decomposition of these composites occurs at wide temperatures ranging from 230 to 650 °C. This profile can be understood by combining the small changes around 230–310 °C (Stage II) and 310–420 °C (Stage III) with the large changes observed at about 500 °C (Stage IV). Therefore, it is estimated that the effective blackening due to the formation of π-expanded structures is promoted by heat treatment at temperatures above 400 °C. Elemental analysis supports the structural change in the attached polymer by the heat treatment. The CN ratio of the **Sil-P_17_**/H_75_-0 without heat treatment was calculated to be 9.74 from 10.5 and 1.08 wt % in carbon and nitrogen, respectively. This CN value is close to the 9.90 of the **P_h_-3** particles prepared without silica. In contrary, the incubation of **Sil-P_17_**/H_75_-0 at 560 °C evidently increased the CN ratio to 37.3, with a significant decrease in nitrogen. This phenomenon was common to the non-silica polymer particles from DHN and TMTA. Therefore, it is presumed that heat treatment enhanced the thermally induced denitrification reaction, and it developed into a long-range π-conjugated structure to become black via multi-step reactions.

The Raman spectroscopy results support a thermally induced increase in the sp^2^ carbon content. From Figure 5b, it is clear that the incubation of **Sil-P_17_**/H_75_-0 at 560 °C increased the typical absorption intensities around 1590 and 1350 cm^−1^, which were attributed to the G and D bands based on the sp^2^ and sp^3^ carbons, respectively [47,48,49] The spectral pattern and the I_D_/I_G_ ratio (ca. 0.5) indicate that the blackened component on the silica is likely to be amorphous carbon [50]. However, further detailed investigation is needed to determine the exact structure.

### 3.4. Pore Characterization of Black Polymer-Laminated Silica

The maintainability of porosity after polymer lamination is an important requirement for a variety of applications. In this study, the particle characterization of the raw silica, the polymer-laminated silica, and the blackened silica was examined using SEM and Brunauer–Emmett–Teller (BET) analyses. The comparison of **Sil-P_17_**/H_75_-0 and **Sil-P_50_**/H_75_-0 before heat treatment summarized in Table 3 shows that polymerization increased the particle size by decreasing both the pore size and the specific surface area. In particular, **Sil-P_50_**/H_75_-0, which obtained a polymer content of 48.3 wt %, showed an undesired loss of surface area, corresponding to a decrease in the pore volume to 21%.

In contrary, **Sil-P_17_**/H_75_-0, in which 15.2 wt % of the polymer was introduced, maintained a good porosity of 229 m^2^ g^−1^ in their specific surface area, corresponding to 69% of the raw silica. Further improvement was confirmed via heat treatment. In the case of **Sil-P_17_**/H_75_-0, it restored the pore characteristics by slightly decreasing the particle size. For instance, incubation at 200 and 560 °C increased the porosity to 235 and 279 m^2^ g^−1^ in their specific surface areas, which corresponded to 71% and 85% of the raw silica, respectively. Notably, the average pore diameter was larger than that of the raw silica after heat treatment at 560 °C. It is difficult to determine if the original porosity is maintained by thin layer formation on the pore surface or because a newly created polymer layer increases the surface roughness. However, it is noted that the non-silica-supported polymer particles exhibited no significant specific surface area, and when the polymerization with silica was carried out using low concentrations, the resulting surface was smoother than at high concentrations. Therefore, these facts indicate that our method suggests the possibility of maintaining a good initial porosity.

The surface characterization was evaluated via ζ-potential measurement. Figure 6 shows the pH dependencies of the raw silica (**Sil-0**) and the polymer-laminated silica (**Sil-P_17_**/H_75_-0 and **Sil-P_17_**/H_75_-H_200_) on the ζ-potential profiles. From Figure 6, the raw silica exhibited negative values at pHs above 5. Because the bending point is obtained at a pH ranging between 5 and 8, the pH dependency of **Sil-0** can be attributed to the pKa = 5.6 of Si-OH [51]. In contrary, the polymer lamination of silica (**Sil-P_17_**/H_75_-0) resulted in a positive charge under acidic conditions, while the negative charge remained in the alkaline pH. From the estimated structure of the polymer in Figure 1, such an ampholytic property is presumed to be derived from an imino group of TMTA as a cross-linker and a phenolic group of DHN. 

On the other hand, heat treatment at 560 °C (**Sil-P_17_**/H_75_-H_560_) reduced the positive charge in an acidic pH, which can be explained by a thermally induced denitrification reaction to become a carbon-like structure. Looking at the pH profile in detail, the negative charge is seen in a wide range of pHs, but also it seems to be a two-step transition. In addition, we confirmed both the reduction of positive charge in an acidic pH and the remaining of the negative charge in an alkaline pH for the heat treatment of non-silica-supported DHN-TMTA particles. Therefore, the negative charge of **Sil-P_17_**/H_75_-H_560_ in a wide range of pHs indicates the existence of a phenolic OH, although it is indistinguishable from that of a Si-OH moiety from the matrix silica. 

### 3.5. Detection of Specific Interface Property by Selective Adsorption

First, through column methodology, a fundamental property of the adsorptivity and selectivity of the polymer-laminated silica was examined using hydrophobic substances such as alkylbenzene homologues and simple aromatic hydrocarbons such as benzene and naphthalene as elutes. When the **Sil-P_17_**/H_75_-H_200_ and **Sil-P_17_**/H_75_-H_560_ columns were used for the separation of alkylbenzene homologues with alkyl chains of different lengths using polar solvents such as methanol, ethanol, and acetonitrile as the mobile phase, their elution orders were identical to those of octadecylated silica (ODS), which has been mostly used in HPLC. This indicates that the **Sil-P_17_**/H_75_-H_200_ and **Sil-P_17_**/H_75_-H_560_ components in silica can act as a hydrophobic phase. However, a detailed analysis of the retention behavior evokes a new perspective for understanding specific behavior, especially in the **P_17_**/H_75_-H_560_ moiety. For instance, **Sil-P_17_**/H_75_-H_560_ exhibited the fact that the selectivity (α) calculated from each retention factor was 10.7, whereas the ODS was 1.25 when the retention factor of 1-butylbenzene (C_10_H_14_) was compared with that of tert-butylbenzene (C_10_H_14_). Additionally, a similar difference was observed in the comparison of naphthalene (C_10_H_8_) with 1-butylbenzene (C_10_H_14_): α = 26.5 and 1.80 for **Sil-P_17_**/H_75_-H_560_ and ODS, respectively (see Table S1). ODS exhibited small separations because these elutes have the same number of carbon atoms and there is no significant difference in their log P as an indicator of hydrophobicity. Therefore, to explain the unusual selectivity enhancement owing to heat treatment, we should consider a different mechanism that recognizes the other physical properties of elutes. To investigate the specific retentivity and selectivity of **Sil-P_17_**/H_75_-H_560_, geometrical isomers of stilbenes were selected as elute samples. As summarized in Table 4, monomeric ODS shows a small selectivity (α= 1.04) for the *trans-* and *cis-*isomers of stilbenes, whereas a small increase to 1.19 can be observed in polymeric ODS. These small values are because the isomers have no significant hydrophobicity difference, which can be characterized by their log P = 4.71 and 4.81 in the *cis*- and *trans-*forms, respectively. In contrast, a higher selectivity (α = 1.68–3.65) can be observed for the special separating agents that are expected to have additional interaction mechanisms, such as the carbonyl–π [15,19,52], π–π [53,54,55,56], and polar–π [57,58] interactions.

Non-heat-treated **Sil-P_17_**/H_75_-0 exhibited a high selectivity (α = 3.60), which corresponded to the highest values obtained with the special adsorbents listed in Table 4. No significant increase in retentivity (*k’*) and selectivity (α) was observed via heat treatment at 200 °C. In contrary, heat treatment at 560 °C brought a remarkable increase in the retentivity. Using the same solvent system for **Sil-P_17_**/H_75_-0, both the isomers were not eluted within 2 h. If this system was applied for polycyclic aromatic hydrocarbons such as anthracene and more π-electron-rich substances, it would be difficult to elute them. We successfully detected the elution of stilbenes for the calculation of selectivity by adjusting the polarity of the mobile phase. Thus, we observed the α value of 14.0 in acetonitrile. Notably, this value is the highest. According to our previous studies, multiple interactions between the adsorbent and solute increase the retentivity and selectivity, even if it is based on weak interactions such as the π–π interaction. In the case of stilbenes, it is understandable that **Sil-P_17_**/H_75_-H_560_ with carbon-like π-electron planes promote multiple interactions with *trans*-stilbene with a planar structure that is much stronger than that of a non-planar cis-stilbene.

## 4. Conclusions

In this study, we have presented a versatile method for introducing a carbon-like π-electron-rich organic phase into porous silica. Our method can be emphasized by its applicability for on-site polymerization without the pre-modification of the carrier particle surface, the consequent facile carbonization process, and the good maintainability of the initial porosity of the carrier particles. These advantages are derived from a bottom-up creation method for a carbon structure where the small key molecules are adsorbed into the pores. In this study, we focused on the application of high-selective adsorption using the surface modification of porous silica. However, because of the change in monomers and carriers, our method experienced limited applicability. For instance, through a preliminary test we have confirmed that fluorescent particles can be created by changing the monomer from 1,5- to 2,6-DHN as a starting monomer. Moreover, other applications include the modification of the catalyst surface, masking, and stability enhancement. We hope to report these findings in the near future.

## Figures and Tables

**Table 1:** Polymerization conditions and particle characterization.

Abbr.	Method	Solvent (Vol %)				Conc. (mM)	Reaction Temp. (°C)	Reaction Time	Average Particle Diameter (μm)	CV (%)
		EtOH	H_2_O	THF	IPA					
**P_m_-1**	Microwave	-	30	70	-	10	150	3 min	0.18	33
**P_m_-2**	Microwave	100	-	-	-	30	150	3 min	0.69	18
**P_h_-1**	Heating	100	-	-	-	1	75	24 h	0.31	19
**P_h_-2**	Heating	100	-	-	-	10	75	12 h	0.92	10
**P_h_-3**	Heating	100	-	-	-	30	75	6 h	1.33	7
**P_h_-4**	Heating	50	-	-	50	150	75	6 h	2.06	6
**P_h_-5**	Heating	100	-	-	-	150	75	6 h	2.41	25

**Table 2:** The reaction conditions for the polymer lamination and heat treatment for blackening.

Abbrev. ^a^	Process I: Polymerization ^b^				Process II: Heat Treatment ^c^			
	Method	Initial wt. ratio (%)	Temp. (°C)	Time	Method	Medium	Temp. (°C)	Time
Sil-0 ^d^	–	–	–	–	–	–	–	–
Sil-P_14_/M_100_-0	Microwave	14	100	3 min	–	–	–	–
Sil-P_14_/M_100_-M_200_	Microwave	14	100	3 min	Microwave	in EG	200	10 min
Sil-P_14_/M_100_-M_300_	Microwave	14	100	3 min	Microwave	in EG	300	10 min
Sil-P_17_/H_75_-0	Heating	17	75	4 h	–	–	–	–
Sil-P_17_/H_75_-H_200_	Heating	17	75	4 h	Heating	under N_2_	200	2 h
Sil-P_17_/H_75_-H_400_	Heating	17	75	4 h	Heating	under N_2_	400	2 h
Sil-P_17_/H_75_-H_200_	Heating	17	75	4 h	Heating	under N_2_	560	2 h
Sil-P_17_/H_75_-H_900_	Heating	17	75	4 h	Heating	under N_2_	900	2 h
Sil-P_50_/H_75_-0	Heating	50	75	4 h	–	–	–	–

a: The abbreviations in parentheses exhibit the method and temperature in the processes I and II, where M or H indicate the methods with and without microwaves, respectively. b: Ethanol was used as a solvent in process I. The initial wt. ratio corresponds to the weight ratio of the monomers for silica. c: In the microwave method, ethylene glycol (EG) was used as the suspension solvent. d: **Sil-0** indicates the raw porous silica.

**Table 3:** Particle characterization after the polymer lamination and heat treatment.

	Polymer (wt %)	Diameter (μm)	PS (nm)	SA (m^2^g^−1^)	PV (mL g^−1^)
**Sil-0**	–	4.37	12.0	330	1.01
**Sil-P_17_**/H_75_-0	15.2	4.65	9.7	229	0.69
**Sil-P_50_**/H_75_-0	48.3	5.02	6.6	124	0.21
**Sil-P_17_**_/_H_75_-H_200_	15.0	4.48	9.7	235	0.72
**Sil-P_17_**/H_75_-H_560_	9.83	4.42	12.5	279	0.75

Polymer: content (wt %) of organic component for silica determined by TGA. Diameter: average diameter (μm) of particles determined via SEM. PS: average pore size (nm); SA: specific surface area (m^2^g^−1^); and PV: pore volume (mg L^−1^) were determined by BET.

**Table 4:** Comparison of the geometrical selectivity for the isomers of stilbenes with various adsorbents.

Separation Agents	Organic Phase ^a^	Mobile Phase		α ^b^	*k*′ for Stilbenes		Note
		Solvent	Temp.		*trans*	*cis*	
**Sil-P_17_/**H_75_-H_560_	p	CH_3_CN only	20 °C	**14.0**	13.7	0.98	This work
**Sil-P_17_/**H_75_-H_560_	p	EtOH:THF (8:2)	20 °C	**9.80**	1.96	0.20	This work
**Sil-P_17_/**H_75_-H_560_	p	EtOH only	20 °C	incal. ^c^	not eluted	4.53	This work
**Sil-P_17_/**H_75_-H_560_	p	MeOH:H_2_O (9:1)	20 °C	incal. ^c^	not eluted	not eluted	This work
**Sil-P_17_**/H_75_-H_200_	p	MeOH:H_2_O (9:1)	20 °C	**3.67**	1.77	0.48	This work
**Sil-P_17_/**H_75_-0	p	MeOH:H_2_O (9:1)	20 °C	**3.60**	1.60	0.62	This work
ODS-m	m	MeOH:H_2_O (9:1)	25 °C	1.04	0.72	0.69	Ref [52]
ODS-p	p	MeOH:H_2_O (9:1)	25 °C	1.19	3.05	2.57	Ref [53]
MA-based	p	MeOH:H_2_O (7:3)	25 °C	1.97	5.51	2.80	Ref [52]
ODA-based	p	MeOH:H_2_O (7:3)	15 °C	2.34	7.47	3.19	Ref [52]
poly(L-Alanine)	p	MeOH:H_2_O (6:4)	20 °C	1.84	1.42	0.77	Ref [15]
Glutamide-based	m	MeOH:H_2_O (7:3)	20 °C	3.04	n.d. ^d^	n.d. ^d^	Ref [19]
CN-based	p	MeOH:H_2_O (7:3)	25 °C	2.25	0.74	0.33	Ref [54]
Pyridine-based	p	MeOH:H_2_O (9:1)	35 °C	2.28	0.41	0.18	Ref [55]
Carbazole-based	p	MeOH:H_2_O (9:1)	35 °C	1.79	1.59	0.89	Ref [56]
Im^+^-based IL	p	MeOH:H_2_O (6:4)	25 °C	1.68	0.69	0.41	Ref [57]
Im^+^-based IL/MO	p	MeOH only	25 °C	3.65	n.d. ^d^	n.d. ^d^	Ref [58]

^a^ “p” and “m” indicate polymeric and monomeric types, respectively. ^b^ Calculated by the ratio of *k′* of *trans*- to *cis*-isomers. ^c^ Incalculable. ^d^ No data.

## Supplementary Materials

The following are available online at https://www.mdpi.com/2079-4991/10/9/1882/s1: Figure S1: Time courses of the diameter change of the particles generated in the polymerization of DHN with TMTA in ethanol at 75 °C. Figure S2: Time course of the carbon-nitrogen (CN) ratio of the products determined by elemental analysis, and the estimated structures of precursors and polymers as possible intermediates in the reaction mixture. Figure S3: UV-visible and fluorescence spectra monitored during the polymerization. Figure S4: Thermal decomposition profiles of **Sil-P_17_** in air. Table S1: Separation factors (α) of hydrocarbons with C_10_-hydrocarbons with ODS and **Sil-P_17_**/H_75_-H_560_ columns.
