# Supplementary material for: Fabrication of Carbon-Like, π-Conjugated Organic Layer on a Nano-Porous Silica Surface
Source: Nanomaterials (Basel). 2020 Sep 20;10(9):1882. doi: 10.3390/nano10091882 (PMC7558701; doi:10.3390/nano10091882)
Supplement: Supplementary file 1 [file nanomaterials-10-01882-s001.pdf]

Table of Contents

Figure S1. Time courses of the diameter change of the particles generated in the polymerization of DHN with TMTA in ethanol at 75 ˚C.

Figure S2. Time course of the carbon-nitrogen (CN) ratio of the products determined by elemental analysis, and the estimated structures of precursors and polymers as possible intermediates in the reaction mixture.

Figure S3. UV-visible and fluorescence spectra monitored during the polymerization.

Figure S4. Thermal decomposition profiles of **Sil-P$_{17}$** in air.

Table S1. Separation factors (α) of hydrocarbons with C_{10}-hydrocarbons with ODS and **Sil-P$_{17}$**/H_{75}-H_{560} columns.

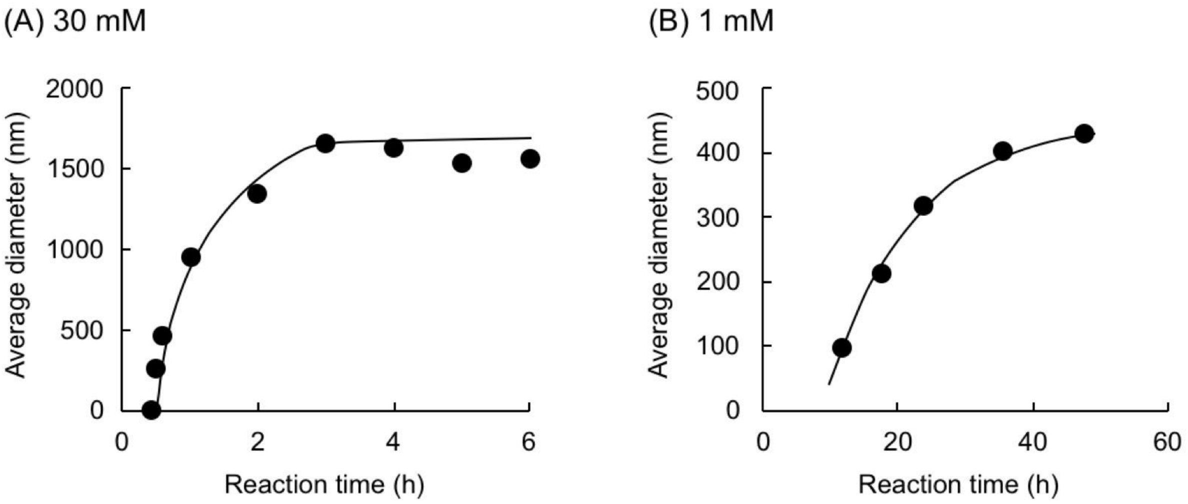

Figure S1. Time courses of the diameter change of the particles generated in the polymerization of DHN with TMTA in ethanol at 75 ˚C. The initial concentrations: (A) DHN = TMTA = 30 mM; (B) DHN = TMTA = 1 mM. The particle diameter was monitored by DLS.

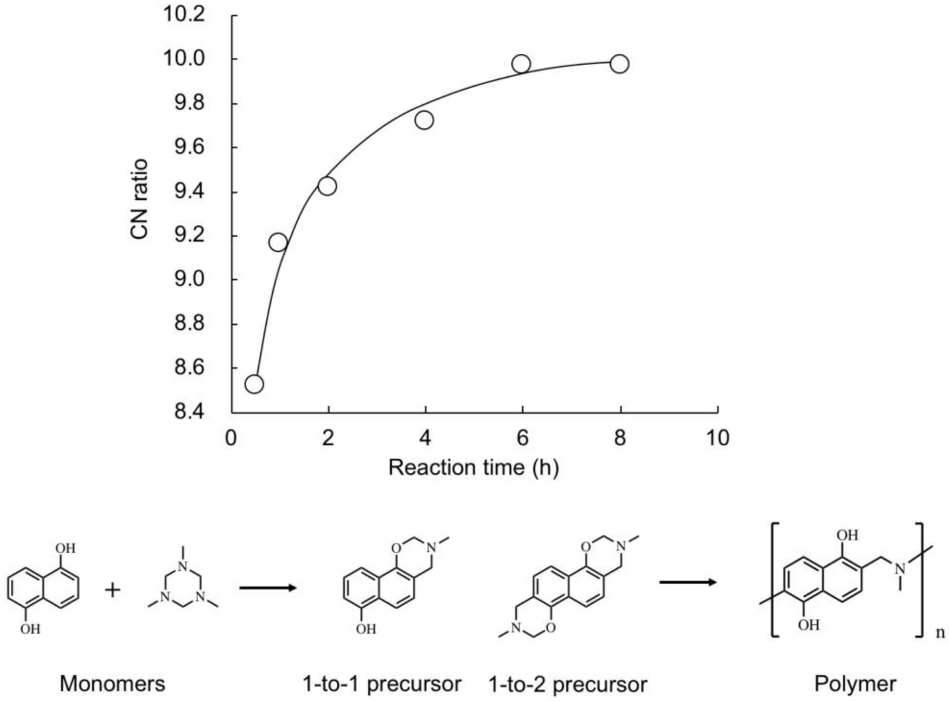

Figure S2. Time course of the carbon and nitrogen content (CN) ratio of the products determined by elemental analysis, and the estimated structures of precursors and polymers as possible intermediates in the reaction mixture. The polymerization condition is shown in **Sil-P**$_{17}$/H$_{75}$-0 in Table 1.

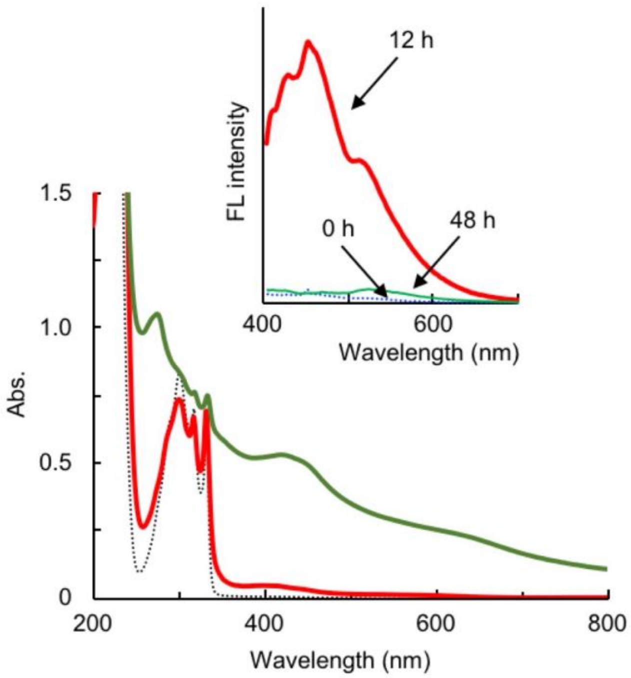

Figure S3. UV-visible and fluorescence spectra monitored during the polymerization. The reaction condition is shown in $\mathbf{P_h}$**-1** in Table 1.

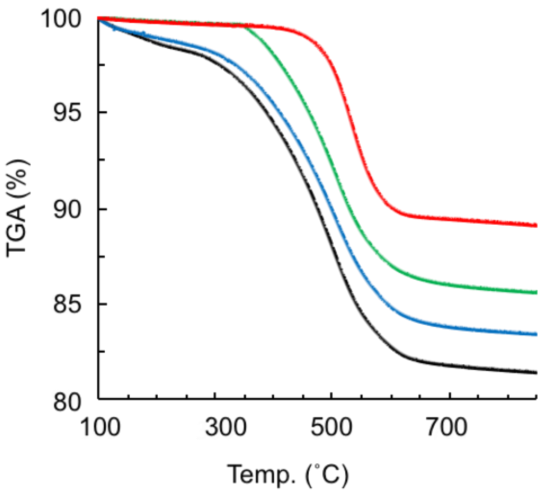

Figure S4. Thermal decomposition profiles of $\mathbf{Sil\text{-}P_{17}}$ in air. The black, blue, green and red lines indicate $\mathbf{Sil\text{-}P_{17}}$/$H_{75}$-0, $\mathbf{Sil\text{-}P_{17}}$/$H_{75}$-$H_{200}$, $\mathbf{Sil\text{-}P_{17}}$/$H_{75}$-$H_{400}$, and $\mathbf{Sil\text{-}P_{17}}$/$H_{75}$-$H_{560}$, respectively.

Table S1. Separation factors (α) of hydrocarbons with C_{10}-hydrocarbons with ODS and **Sil-P$_{17}$**/H_{75}-H_{560} columns.

Samples			ODS		**Sil-P$_{17}$**/H_{75}-H_{560}	
Names and structures		log *P*	*K′*	α	*k′*	α
1-Butylbenzene	$C_{10}H_8$	4.51	2.17	1.25	2.76	10.6
tert-Butylbenzene	$C_{10}H_{14}$	4.10	1.73		0.26	
1-Butylbenzene	$C_{10}H_8$	4.51	2.17	1.80	2.76	26.5
Naphthalene	$C_{10}H_8$	3.16	4.29		6.89	

Mobile phase: CH_3CN/H_2O (v/v) = 70/30 for ODS. Flow rate: 1.0 mL min^{-1}.
Column temperature: 20 ˚C.
